# Author Correction: Critical shifts in lipid metabolism promote megakaryocyte differentiation and proplatelet formation

**DOI:** 10.1038/s44161-023-00389-6

**Published:** 2023-11-16

**Authors:** Bianca de Jonckheere, Ferdinand Kollotzek, Patrick Münzer, Vanessa Göb, Melina Fischer, Kristina Mott, Cristina Coman, Nina Nicole Troppmair, Mailin-Christin Manke, Monika Zdanyte, Tobias Harm, Manuel Sigle, Dominik Kopczynski, Andrea Bileck, Christopher Gerner, Nils Hoffmann, David Heinzmann, Alice Assinger, Meinrad Gawaz, David Stegner, Harald Schulze, Oliver Borst, Robert Ahrends

**Affiliations:** 1 Institute of Analytical Chemistry, University of Vienna, Vienna, Austria; 2 Vienna Doctoral School in Chemistry, University of Vienna, Vienna, Austria; 3 DFG Heisenberg Group Cardiovascular Thromboinflammation and Translational Thrombocardiology, University of Tübingen, Tübingen, Germany; 4 Department of Cardiology and Angiology, University of Tübingen, Vienna, Germany; 5 Institute for Experimental Biomedicine, University Hospital Würzburg, Würzburg, Germany; 6 Rudolf Virchow Center for Integrative and Translational Bioimaging, Würzburg, Germany; 7 Joint Metabolome Facility, University of Vienna and Medical University of Vienna, Vienna, Austria; 8 Forschungszentrum Jülich GmbH, Institute of Bio- and Geosciences (IBG-5) Jülich, Jülich, Germany; 9 Institute of Physiology, Centre of Physiology and Pharmacology, Medical University of Vienna, Vienna, Austria

**Keywords:** Haematopoietic stem cells, Lipidomics, Proteomics, Mass spectrometry

Correction to: *Nature Cardiovascular Research* https://doi.org/10.1038/s44161-023-00325-8, published online 31 August 2023.

In the version of this article initially published, the Acknowledgements section omitted mention of funding from the Austrian Science Fund, FWF DMP I6303. The error has been corrected in the HTML and PDF versions of the article.

